# Satisfaction with care among patients with non-metastatic breast cancer: development and first steps of validation of the REPERES-60 questionnaire

**DOI:** 10.1186/1471-2407-7-129

**Published:** 2007-07-16

**Authors:** Gautier Defossez, Simone Mathoulin-Pelissier, Isabelle Ingrand, Isabelle Gasquet, Lynda Sifer-Riviere, Pierre Ingrand, Roger Salamon, Virginie Migeot, the REPERES research network

**Affiliations:** 1 University Hospital and University Institute of Public Health, Poitiers, France; 2 Cancer Aquitaine Network and Bergonié Institute, Centre Régional de Lutte contre le Cancer Sud-Ouest, Bordeaux, France; 3 INSERM U669 and Assistance Publique – Hôpitaux de Paris, Direction de la Politique médicale, Paris, France; 4 CERMES, INSERM, Ecole des Hautes Etudes en Sciences Sociales, Paris, France; 5 Unité INSERM 593, Institut de Santé Publique Épidémiologie et Développement, Bordeaux, France

## Abstract

**Background:**

The care itinerary for cancer involves difficulties that occur in several different areas, whether in the diagnostic procedures, in surgery, or in adjuvant treatment. The aim of this work was to obtain a valid instrument measuring satisfaction among patients with breast cancer and exploring their care itinerary overall.

**Methods:**

*Development phase*: Patient focus groups were implemented in two French regions in order to identify areas of satisfaction in relation to the different phases of care provision in breast cancer. On the basis of the literature and the themes and wordings derived from the focus groups, the patients identified several areas of satisfaction, which they found to be partially covered in an American satisfaction measure that has been validated in the French general population (the Consumer Satisfaction Survey in its French version, CSS-VF, 39 items). The patient focus groups suggested adaptation of certain dimensions of this instrument to the potential care providers (37 items) and produced 45 new items in six areas.

*Validation phase*: Using a large sample of patients (cohort of 820 women with invasive non-metastatic breast cancer) approached one month after treatment, this phase selected items that were comprehensible (non-response rate < 10%), non-redundant (r < 0.80) and reproducible (test-retest conducted on a sub-sample of 166 patients). The dimensions were identified by factor analysis on the selected items. Divergent and discriminant validity were assessed (relationships with quality of life questionnaire, comparisons between extreme groups).

**Results:**

Results were in favour of not inserting additional broken-down items into the CSS-VF and retaining 21 new items. The factor analysis found the initial structure of the CSS-VF (39 items in 9 dimensions) and the 21 new items divide up into four dimensions (listening abilities and information provided by doctors, organisation and follow-up of medical care provision, psychological support, material environment). No redundancy was observed between new items and CSS-VF items. Internal consistency was high. Divergent and discriminant validity were satisfactory.

**Conclusion:**

Adding four new dimensions to the CSS-VF yielded a valid 60-item instrument for assessment of care provided in breast cancer. These promising results now require further investigations of its responsiveness and its robustness in other linguistic, cultural and healthcare settings.

## Background

Breast cancer is the most frequent cancer among women, with a little over one million new cases per year worldwide [1]. Over recent years, the way the disease has been catered for has changed (screening, diagnosis, prognostic markers, new surgical techniques, oral treatment, etc). As a result, prognosis has improved, and patients have a more regular follow-up by physicians. Alongside this, the assessment of patient satisfaction has gained ground in the literature on cancer. This can be explained by the importance given to patient preferences in medical decisions, and also by the need to measure the results of health strategies [2,3]. Thus today measuring satisfaction among patients with non-metastatic breast cancer over the complete the care itinerary is an essential step in improving the way the pathology is catered for [3-6].

Satisfaction is a concept that is at once theoretical, multidimensional, and subjective. This concept, which cannot be measured by direct observation of the care provided, involves the identification of expectations, needs, perceptions, past experiences, opinions and attitudes of patients [4,7,8]. Several authors have thus considered that the assessment of satisfaction required an operational formalisation of the concept into dimensions with their constituent items making up questionnaires [7], and hence this rapidly entailed the need to assess the psychometric properties of such instruments [9].

The majority of these instruments were developed in the USA or the United Kingdom, and socio-cultural differences or differences in the health systems restricted their use in assessment of care itineraries in other countries. In addition, many of these questionnaires measure satisfaction in hospitalised patients, while others focus on a particular moment in the care itinerary, such as the consultation [10-17]. Finally, some questionnaires do not possess the required psychometric properties [18,19].

The care itinerary for breast cancer involves difficulties that occur in several different areas, whether in the radiological and histological diagnostic procedures, in surgery, or in complementary treatment. Pluri-disciplinarity is essential in the therapeutic decision, and this entails coordination of care interventions throughout treatment, as well as between the different phases of treatment, and subsequent to treatment. The range of expertise required leads to time lapses, and complicates the organisation of care. This applies to all the actors in the care itinerary, and the time required to reach collegiate decisions can have a negative effect on patients. Patients expect care to be instated promptly, and to be accompanied by clear information and psychological support.

To our knowledge, no satisfaction questionnaire explores patient opinion in the different phases of treatment for breast cancer, while several areas of satisfaction have quite specific links with these particular care itineraries.

The aim of the study was therefore to develop a questionnaire measuring satisfaction with the care itinerary as a whole among patients with non-metastatic breast cancer subsequent to initial treatment. In view of the repeated developments of new satisfaction questionnaires, and the time required to construct and validate them [20,21], we decided to start from an existing validated satisfaction questionnaire, the Consumer Satisfaction Survey in its French version [22,23].

## Methods

This study was conducted in two French administrative regions (Aquitaine and Poitou-Charentes) representing 7 to 8% of the national population of France. This study is integrated into a general project of evaluative research on the performance of health networks, entitled REPERES (Recherche Evaluative sur la Performance des Réseaux de Santé).

**Figure 1:**
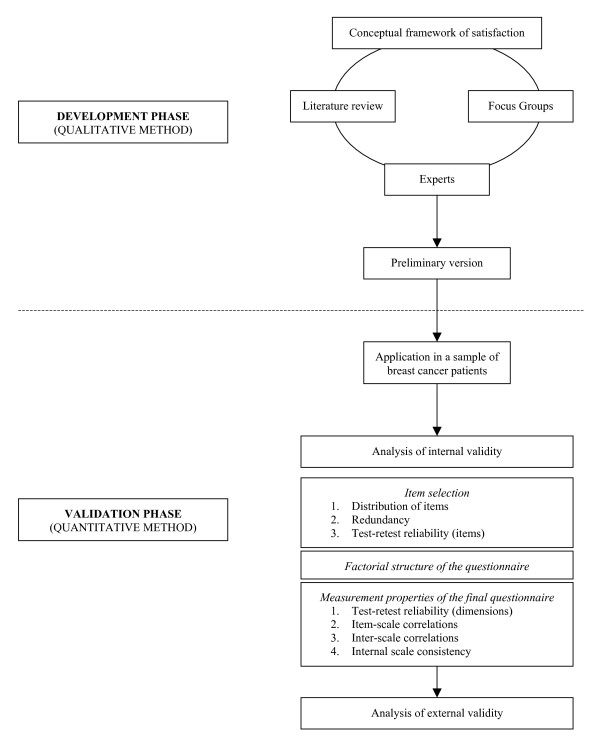
Simplified development and validation process of the REPERES-60 questionnaire.

This article reports on the two phases of the development and validation of this questionnaire (Figure 1):

1. The first phase (qualitative method) was conducted to generate items and identify domains, using a literature review and focus groups involving breast cancer patients;

2. The second phase (quantitative method) was the first steps of a validation process designed to identify the best set of items using psychometric and statistics methods, and to analyse the association or correlation with other external indicators and assess the known-group validity of the new tool.

### Development of the questionnaire (qualitative method)

Two focus groups were organised successively, involving fifteen patients with cancer for each focus group. The patients were selected to ensure diversity within the focus group, both for clinical and socio-demographic characteristics and for care itinerary [see Additional file 1]. The mean age of patients was 62 years (range 40 to 78), and six were retired. All women were some time from their initial treatment, and were either in remission or in relapse for some cases.

The first group interviews aimed to stimulate participants to put their personal experiences into words, and to explore their perception of quality in the care process [24]. The interviews, recorded with the agreement of the participants, were intended to collect the wordings used by non-medical individuals to talk about certain medical notions. A review of the literature had been conducted to find seven published questionnaires [10,11,13,14,22,23,25,26], which enabled the drafting of a list of satisfaction dimensions which was presented to the focus group patients. The main domains covered in the questionnaires reviewed were fairly classic: human relationships, technical quality of care, accessibility and convenience, economic aspects, efficiency of care, continuity of care, material environment and availability of doctors [7,19,27]. The patients in the focus group successively discussed the different phases in care and treatment of breast cancer.

A list of elements for item development (themes and wordings) derived from the verbatim transcribed from the first focus groups was developed by the REPERES work group (sociologists who analysed content).

On the basis of this material, the patients in the second focus group identified several areas of satisfaction, which they found to be covered in a questionnaire developed by Davies & Ware [22] and which has been culturally adapted and validated in French (CSS-VF) [23].

The second focus group made the choice of using the 9 generic dimensions of the CSS-VF as the basis of their work. It is true that certain validated satisfaction questionnaires for use among cancer patients such as the EORTC InPatsat-32 cover some of these dimensions [17], but they are restricted to the hospitalisation period of the patients' care itineraries. In addition, the CSS-VF had the advantage of exploring satisfaction with General Pratitionner (GP) and specialist care separately: this was indeed used by the focus group to adapt certain dimensions to the different health care actors involved in care of breast cancer, adding 37 items. Thus the items concerning the GP were completed by two items for the gynaecologist and the radiologist, and the items concerning the specialist were subdivided into two items, for the surgeon and the oncologist. This new structure made it possible to distinguish between primary care, provided routinely for diagnosis and follow-p by the GP, the gynaecologist and the radiologist, and secondary care providing treatment of the cancer and hence implicating the surgeon and the oncologist.

However several issues in the list derived from the verbatim were not covered in the CSS-VF, and they were discussed and worded as items by the patients, with methodological support from a sociologist. The patients in the focus groups put particular emphasis on three main themes:

- Psychological support, required in the course of the different phases of treatment: in screening procedures or at the time of the clinical identification of a tumour, when "sudden psychological pressure sets in", while waiting for results which are "a big source of anxiety", and at the time when patients leave the medical environment and return to work or everyday life, where they gain "awareness" of the illness, a time when there is a "very great feeling of isolation", whether medical, psychological geographical or family;

- Listening abilities of doctors and the information they provide: the importance for patients of someone who will "listen", who will enable a "person-to person relationship"; the importance of being "informed and accompanied right up to the moment when the consequence of surgery and the scars are confronted", of being able to "talk about treatment and its side effects", of being able " to talk about breast reconstruction surgery early on" and about "what information to give the close circle of family or friends";

- The organisation of follow-up after treatment: patients emphasise " the length and the succession of treatment phases which is more difficult to cope with than the treatment itself", the need to "have a part in the choice of treatment and mode of care", the importance of feeling that "staff work as a team ", and that "communication between the GP and the specialist works well".

The quality of the material environment in which cancer is cared for and treated was also discussed in the focus groups, and although the theme is present in the literature, the aspect of the confidentiality afforded by premises is not in fact explored. The themes of efficiency of care and access to medical information were developed independently by the REPERES working team, in response to the fact that the issues of access to medical files was an ongoing debate at the time (cancer plan) and also to the procedures for evaluation of efficiency of care that were being set up.

Three new items were drafted to complete the existing dimension on insurance cover and 42 new items were drafted to fall into five areas of satisfaction: 1) listening abilities and information provided by the doctors; 2) organisation and follow-up of medical care provision; 3) psycho-social support; 4) outcomes of care; 5) and material environment.

The format of the response choices was ordinal, in line with that used for the CSS-VF, and the best suited to the positive response trends often observed in satisfaction surveys [7,28].

The test version of the full questionnaire, administered in the form of a self-assessment, comprised a total of 121 items: 45 new items and 76 derived from the original CSS-VF. This version of the questionnaire was then tested with a population of breast cancer patients to assess understanding and exhaustiveness of the items, and this led to some minor alterations to the questionnaire.

### Validation of the questionnaire (quantitative method)

#### Sample and data collection

A cohort survey was conducted from October 2003 in public and private cancer centres. The patients included in the cohort complied with the following criteria: 1) a first diagnosis of invasive non-metastatic breast cancer in the course of 2003; 2) at least two contacts for cancer with one of the health professionals in one of the two regions between diagnosis and the first year of follow-up; 3) signed informed consent.

The satisfaction questionnaires were sent at three moments in the care itinerary [29]: one month after the end of the initial treatment for the study of the psychometric properties of the instrument (this involved the whole cohort, n = 975); then a year later, and again a week after that to study reproducibility of items (this involved a sub-group representative of the cohort obtained by random selection, n = 172). The reproducibility study entailed questioning patients at a stable period in the course of their disease, and no psychometric study was performed at one year.

The logistics of data collection were ensured by a team specifically dedicated the project, and involved recall procedures at each stage in the study. Telephone support was established to provide patients with explanations or complementary information on the aims, design and expected results of the study. The process of patient inclusion as a whole had been previously approved by the consultative committee on the processing of information in medical research of CNIL (French national commission on individual privacy).

Responses provided to the questionnaires were captured using the Data Base Management System 4D Server, and data were analysed using the SAS programme, version 8.2. (Windows). A verification procedure was implemented via independent duplicate data capture to check the whole data set.

Finally questionnaires that presented more than half the responses missing were removed from the statistical analysis.

#### Analysis of internal validity

##### Item selection

The selection of items was based mainly on clinical criteria and priorities expressed by patients in the focus groups. The internal validity of the 121 items was studied by successively examining the distribution of items, any redundancy, and their reproducibility over time.

##### Distribution of items

The distribution of each item was studied to see whether the full range of response choices was used, in order to detect items that were not sufficiently informative because the variability of response was insufficient: floor and ceiling effects. An item was removed if one of the response choices received more than 50% of the responses provided [30]. The proportion of missing data was calculated for each item, and this enabled assessment of how well understood or how relevant the questions were. A percentage of missing data on an item under 10% was considered acceptable.

##### Redundancy

Items that were strongly correlated, thus likely to be providing the same information, were looked for. Redundancy between two questions was suspected if the Pearson correlation coefficient was over 0.80 in the inter-item correlation matrix [31,32].

##### Test-retest reliability

The reproducibility of the measure was studied item by item, examining the agreement between measures at the time of the two re-administrations of the questionnaire. As the response format was ordinal, agreement was explored using a weighted kappa coefficient (Fleiss-Cohen weighting) taking into account the degree of disagreement [33]. A kappa coefficient over 0.70 was considered to indicate good reproducibility, it was considered moderate when values were between 0.50 and 0.70, and insufficient below 0.50.

The selection of items took other factors into account: 1) the proportion of non-response: if higher than 10% a problem of acceptability was likely for the item, without systematic exclusion at this stage; 2) the number of items considered sufficient to account for a given dimension: if at least half the items of a dimension were completed for the calculation of the score, the choice was made to keep the item, so as to retain the information in the particular dimension.

##### Factorial structure of the questionnaire

The factorial structure was studied using Principal Component Analysis (PCA). The number of factors retained was determined from eigenvalues greater than or equal to 1 [9]. Factor analysis with rotation enabled identification of the dimensions in the questionnaire, and showed that the items selected measured domains that were identical to those hypothesised at the outset. Rotation was applied to transform the original principal components produced, to ease interpretation. This method searches for a linear combination of the original measurements aiming to maximize the variance of the components loadings. A Varimax rotation (orthogonal) was preferred at a Promax rotation (oblique) if low correlations were observed between factors [34]. An item was attributed to a single factor if it loaded 0.45 or more on a single factor.

##### Properties of the final instrument: reproducibility, item-scale, and inter-scale correlation analyses, scale consistency

The score of each dimension was calculated from the sum of items, standardised so as to obtain a score between 0 (low satisfaction) and 100 (high satisfaction), according to the methodology proposed by Davies and Ware [22]. The score was computed if at least 50% of the items in the dimension were completed. Descriptive statistics were produced for each of the dimensions (mean, standard deviation, percentiles, saturation and asymmetry coefficient).

Reproducibility was studied for the different dimensions using the intra-class correlation coefficient, calculated from a 2-factor variance analysis in a random effect model [35]. An intra-class correlation coefficient over 0.70 was considered to indicate good reproducibility.

The analysis of item-score correlations looked for correlations greater than 0.40 between items and their own dimension. The correlation coefficients should be higher between the item and the dimension to which it belongs (calculating the score without the item under consideration) than between that item and the other dimensions. An analysis of the inter-score correlation matrix was conducted to ascertain independence of dimensions for correlation coefficients below 0.70. The Cronbach alpha coefficient was calculated to assess internal consistency of each dimension. A coefficient over 0.80 was considered satisfactory [32]. Floor and ceiling effects for each dimension were calculated according to the percentage of subjects presenting respectively the maximum and minimum scores in the dimension considered.

#### Analysis of external validity

In a first approach, divergent validity was checked by assessing the relationship between the REPERES-60 scales and the EORTC QLQ-C30 scales and single items (European Organization for Research and Treatment of Cancer Quality-of-Life questionnaire) [36]. Health Related Quality Of Life (HRQOL) summarizes dimensions of the quality of life which reflect the negative functional repercussions (on the physical activity, the psychological state, the social relations) of its disease and its treatment, such as the patient perceives them [37]. Thus these two measures are intended to explore two distinct concepts and their scales and items should not show marked correlation (Pearson's r < 0.40).

In the second approach, the ability of the questionnaire to discriminate between extreme groups was assessed by comparing patient groups that were expected to differ strongly in terms of satisfaction scores. A lower satisfaction level was expected for younger patients (cut-off at the sample median age of 58), patients with higher education (versus primary and secondary education), those who had experienced problems of communication at the time of the announcement of the diagnosis, and patients who reported a poor health status (score less than five on the QLQ-C30 item: "How would you rate your overall health during the past week ?", scored from very poor to excellent) [38-40]. Discriminant validity was investigated using non-parametric tests (Wilcoxon test) [41]. We could estimated, because of none references are existing on this topic, that a five point difference score between extremes groups are representing a significant discrimination.

## Results

### Sample characteristics

Among the 975 patients included, 850 (87%) returned the initial questionnaire within a median time lapse of 18 days. For the study of reproducibility of items, 172 out of 182 (95%) returned the two questionnaires one year after the first completion and again a week after this dispatch.

The proportion of exploitable questionnaires, which presented less than half the responses missing, was over 95% (820/850 and 166/172). Average patient age was 58.0 years (SD 12.4), the majority were living with a partner or spouse (55%) and 44% were working (Table 1).

**Table 1:** Sociodemographical characteristics of breast cancer patients who completed satisfaction questionnaires (less than 50% missing data)

	One month after initial treatment N = 820	One year later and a week after N = 166
Age years (mean ± SD)	58.0 ± 12.4	58.9 ± 12.3

Regional distribution		
Aquitaine area	506 (61.7%)	98 (59.0%)
Poitou-Charentes area	314 (38.3%)	68 (41.0%)

Living with spouse or partner		
Yes	452 (55.1%)	102 (61.5%)
No	368 (44.9%)	64 (38.5%)

Educational level	MD = 18	MD = 3
Primary	190 (23.8%)	46 (28.2%)
Lower secondary	88 (11.0%)	18 (11.0%)
Upper secondary	313 (39.2%)	66 (40.5%)
Higher education	125 (15.7%)	23 (14.1%)
None	82 (10.3%)	10 (6.2%)

Professional status	MD = 14	MD = 4
Working	352 (43.7%)	69 (42.6%)
Retired	329 (40.8%)	62 (38.3%)
Homemaker	81 (10.0%)	24 (14.8%)
Job-seeker	29 (3.6%)	4 (2.5%)
Other	15 (1.9%)	3 (1.9%)

N = Sample size

MD = Missing Data

### Internal validity

#### Item selection [see Additional file 2]

##### Distribution of items

Just one item from the original CSS-VF (Q7) had a high rate of non-response. The replicated (broken-down) CSS-VF items relating to the gynaecologist and the radiologist did not generate adequate response. There was one phenomenon of saturation of response choices on one item of the global satisfaction domain (Q71).

Two of the three items generated to complete the health insurance cover dimension concerned less than one patient in three. The removal of items Q78-Q79 also led to removal of item Q77.

Among the 42 new items, 10 out of the 13 that registered at least 20% non-response were removed from the analysis at this stage. Two items relating specifically to mastectomy or to a particular treatment (Q86 and Q105) were however maintained on account of the importance given to them in the focus group. It was also thought necessary to maintain the third situation-specific item (Q94) in order to explore the care itinerary as a whole, including all aspects of care provision.

Nine new items of the 15 for which the percentage of missing data ranged from 10 to 20% were maintained as far as the factor analysis. This strategy made it possible to have a sufficient and fairly homogeneous number of items for each dimension.

##### Redundancy

High correlations were found for replicated items in the primary care dimensions (the breakdown into GP, gynaecologist and radiologist), and also for the replicated items in the secondary care dimensions (for the surgeon and the oncologist). At this stage these results were in favour of not inserting these additional broken-down items into the CSS-VF, but rather of maintaining the original items. In the subsequent analyses, the broken-down item scores were averaged, retaining solely the distinction between primary and secondary care (Q1 to Q63).

Ten of the 42 new items were redundant, but only the item on information given to understand the treatment (Q80) was removed. The other items were retained to enable the measure to give an account of certain specific sequences in the patients' care itineraries: the time lapses before receiving treatment (chemotherapy, radiotherapy, surgery: r = 0.80–0.82), the quality of consultation premises and respect for privacy in consultations (hospitalisation versus consultation: r = 0.87).

No redundancy was evidenced between items in the CSS-VF and the new items (r ≤ 0.68).

At the end of this stage, 64 items remained out of the 121 items (39 items derived from the CSS-VF and 25 new items).

##### Test-retest reliability

Reproducibility was satisfactory for ten of the 13 (Q64 to Q76) remaining CSS-VF items (weighted kappa ≥ 0.70 for 4 items and between 0.60 and 0.69 for 6 others) and for 22 of the 25 new items (weighted kappa ≥ 0.70 for 11 items and between 0.60 and 0.69 for 11 others). For the six remaining items, the coefficient ranged from 0.55 to 0.59, except for one item in the global satisfaction domain of the original CSS-VF (Q69, kappa = 0.44). No item was excluded on the grounds of its kappa coefficient.

##### Factorial structure [see Additional file 3]

The 64 items retained were entered into principal-components factor analysis with varimax rotation and a twelve-factor PCA solution was retained for 60 items. Four items were not further considered in the subsequent PCA because of absence of loading on any common factor (Q93 to Q96). The factorial structure remained identical with or without them. The first twelve factors explained 45% of the total variance. The dimension "competence" and the dimension "communication skills" for specialists deliberately dissociated in the validation of the French CSS [23] are found here on a single factor (6 items).

The other dimensions of satisfaction were: "access to primary care" (7 items), "access to secondary care" (3 items), "competence and communication skills of primary care doctors", "choice among different doctors" (4 items), "human qualities shown by doctors" (4 items), "global satisfaction" (4 items), "cover for medical expenses" (5 items), "listening abilities and information provided by the doctor" (7 items), "organisation and follow-up of medical care provision" (5 items), "psychosocial support" (5 items) and "material environment" (4 items).

All items had a good loading on their own factor. Only five items had a loading on a secondary component (I5, I20- I22, I54).

##### Properties of the final instrument: reproducibility, item-scale and inter-scale correlation analyses, scale consistency [see Additional file 4]

The reproducibility was very satisfactory for eleven out of thirteen dimensions. The dimensions "access to secondary care" and "competence of doctors in secondary care" had intra-class correlation coefficients of 0.62 and 0.66 respectively.

Correlation coefficients between items and their hypothesised scale were all greater than 0.4. One item out of the 60 (the possibility of obtaining medical information or advice by telephone from the GP and/or the gynaecologist) loaded high on its own scale and also on another scale. One high inter-scale correlation was observed between listening abilities and provision of information by doctors and psychosocial support (r = 0.73). Internal consistency was very high (all Cronbach alpha coefficients ≥ 0.82, except for the global satisfaction score at 0.74).

### External validity [see Additional file 5]

None of the thirteen sub-scales were strongly correlated with the QLQ-C30. The strongest observed correlation was between the QLQ-C30 global quality of life scale and the global satisfaction scale (r = 0.29).

A lower satisfaction level was systematically noted for younger patients, those with higher education, those having experienced problems of communication in the announcement of the diagnosis and those who reported poor health status. All the sub-scales were able to discriminate between patients in terms of age categories, except for the sub-scales competence of secondary care doctors and organisation and follow-up of medical care provision. Differences in satisfaction scores were not significant in relation to educational status in five sub-scales: access to primary care, competence of secondary care doctors, cover for medical expenses, organisation and follow-up of medical care provision and psychological support.

Only the sub-scales for choice among doctors and the cover for medical expenses did not discriminate between patients in terms of health status and problems of communication in announcing the diagnosis.

## Discussion

These first results have shown that the questionnaire REPERES-60 presents very satisfactory psychometric properties. The predominant role given to patients via the focus groups, a procedure often neglected in the early stages of instrument design [6,42], made it possible to explore and develop to the full the content validity of the 21 new items, using dimensions in line with breast cancer patient expectations [10,18,43].

The focus groups demonstrated that the CSS constituted a sound base for the development of complementary dimensions, and also made it possible to reduce the time required to reach a finalised, validated version [5,40]. The CSS presented the required psychometric properties [18], had already been validated in the general population in France [23] and was designed to assess patient opinion with respect to care received, technical competence, human qualities and results. The study of the CSS-VF on a cohort of women with breast cancer made it possible to identify a factorial structure that was strictly similar to that observed in the general population, and thus to validate its metric qualities in a population of cancer patients [18]. The break-down of items relating to each type of medical professional involved in cancer treatment was tested within the CSS-VF. The analysis showed that it was not useful to maintain the distinctions, since the redundancy of the information derived suggested that these items would encumber the instrument for no good reason, and in any case these patients do not all have contact with all the types of professionals that were initially cited. The strong correlations between items can be explained by the way in which cancer is cared for in the French healthcare system. This care is classically provided in one or several health facilities, but with collegiate decisions among the specialists concerned (surgeons and oncologists). A patient when interviewed appears to recall global aspects of care (there is therefore a correlation among specialist scores) rather than aspects concerning each specialist. This tendency is today even more marked in the French "cancer plan" which seeks to develop this collaboration by proposing the establishment of cancer coordination centres.

However, the distinction between primary care professionals (GPs and gynaecologists) and secondary care professionals (surgeons and oncologists) was maintained in the questionnaire [21], as in the initial CSS-VF structure, and enables assessment of the accessibility of the care institution and the continuity of care in ambulatory treatment [42], and issues such as the importance of the informal role of GP in breast cancer follow-up [44].

While the CSS-VF can be used its own, the four new dimensions are complementary to this measure and cannot be evaluated independently. The new measure covers aspects of care that have not been widely dealt with in the literature: continuity of care, solving psychosocial problems [45], or cooperation between oncologists and GP [46]. The information that patients are provided with, and the doctor-patient relationship are areas to which these patients are particularly sensitive. The 21 items integrates the information given on the disease, as well as information on pain management and breast reconstruction surgery. Some items relate specifically to the types of care received by the patient (reconstruction, time-lapse before receiving chemotherapy, radiotherapy and/or surgery). While care was taken to ensure the internal consistency of the new items, the choice was made to maintain certain items that provide information on specific patient treatment that is not otherwise much explored in measures of satisfaction in cancer. "Not applicable" boxes were added to these items to identify patients who were not concerned by the item content.

As expected, lower correlations were observed between the new scales and QLQ-C30 scales, suggesting that conceptually different issues are assessed [21]. If the correlations observed between the two concepts had proved to be high, the results would have been in favour of proximity, rather than independence, between the two tools. The new scales were able to discriminate clearly between patients who differed in terms of health status and communication experiences, and to a lesser degree between patient groups formed on the basis of age and educational status [38-40].

The comparison between the REPERES-60 questionnaire and the main satisfaction questionnaires in the field of cancer was interesting. The literature mainly reports on instruments developed in hospital environments, the most commonly used today being the EORTC IN-PATSAT32 questionnaire. In a hospital environment, the staffs involved are solely on the one hand cancer specialists and on the other the hospital healthcare staff. In a complete care itinerary, all the doctors encountered over the itinerary are involved. This explains why: 1) the enumeration of staff involved in the two questionnaires differs: doctors and nurses as opposed to all the doctors over the full itinerary (primary and secondary care); 2) if the majority of the dimensions explored by the REPERES-60 questionnaire are also found in the IN-PATSAT32 (interpersonal skills, technical skills, information provision, availability, access, exchange of information, comfort, global satisfaction) psychological support, mentioned by the patients as necessary for the announcement of the diagnosis and on leaving the medical environment, is not explored in the IN-PATSAT32.

## Conclusion

Results are convincing but required also further tests such as the responsiveness and the evaluation of its robustness in other settings. It is important in generalizing present results to other linguistic, cultural and healthcare settings [20]. The task now is to analyse the determinants of patient satisfaction in relation to their care itinerary and their clinical characteristics. Identifying the particular needs of breast cancer patients, and pinpointing the aspects of care that are the most important to them, should make it possible to improve the way the disease is catered for, and provide clinicians with indications on the way in which their practice is perceived. In this context, the questionnaire should make it possible, over the complete care itinerary of breast cancer patients, to identify the points where improvements can be implemented and integrated into the organisation of care in cancer. The extension of the questionnaire to other cancers could be envisaged via the implementation of focus groups involving patients with the relevant pathology.

## Competing interests

The author(s) declare that they have no competing interests.

## Authors' contributions

GD performed data analyses and interpretation, and wrote the manuscript. SMP and VM participated in the study design and coordination, and helped to draft the manuscript. II and PI contributed to the data collection, analysis, data interpretation and manuscript preparation. IG, LSR and RS contributed to manuscript preparation. All authors read and approved the final manuscript.

## Pre-publication history

The pre-publication history for this paper can be accessed here:



## Supplementary Material

Additional file 1 Description of focus group members for Aquitaine and Poitou-Charentes regions (qualitative step in development of satisfaction questionnaire). Click here for file

Additional file 2 Floor, ceiling effects, missing data, and weighted kappa coefficients of the preliminary items' questionnaire. Click here for file

Additional file 3 Principal components factor analysis (varimax rotation) computed with the 60 items of the final questionnaire (n = 174). Click here for file

Additional file 4 Results of psychometric tests for the final questionnaire REPERES-60. Click here for file

Additional file 5 Discriminant validity – Extreme group comparisons of mean scores (standard deviations) for the thirteen scales of the REPERES-60 questionnaire. Click here for file

Additional file 6 REPERES-60 questionnaire (the translation is provided solely for the purpose of informing on item content, and is not a validated measure). Click here for file
