# Supplementary material for: Satisfaction with care among patients with non-metastatic breast cancer: development and first steps of validation of the REPERES-60 questionnaire
Source: BMC Cancer. 2007 Jul 16;7:129. doi: 10.1186/1471-2407-7-129 (PMC1933545; doi:10.1186/1471-2407-7-129)
Supplement: Additional file 1 — Description of focus group members for Aquitaine and Poitou-Charentes regions (qualitative step in development of satisfaction questionnaire). [file 1471-2407-7-129-S1.doc]

Description of focus group members for Aquitaine and Poitou-Charentes regions (qualitative step in development of satisfaction questionnaire)

|  |  | Demographic profile | Profession |
| --- | --- | --- | --- |
| Aquitaine | 1 | Female, 78 years | Accountant (retired) |
|  | 2 | Female, 56 years | Teacher (retired) |
|  | 3 | Female, 71 years | Gynaecologist (retired) |
|  | 4 | Female, 50 years | Commercial Management Assistant |
|  | 5 | Female, 52 years | Unskilled |
|  | 6 | Female, 62 years | Executive |
|  | 7 | Female, 58 years | Executive |
|  | 8 | Man, 75 years | Building and public works agent (retired) |
| Poitou-Charentes | 9 | Female, 58 years | Health executive |
|  | 10 | Female, 60 years | Science/engineering graduate (retired) |
|  | 11 | Female, 40 years | Home help |
|  | 12 | Female, 76 years | Social affairs manager (retired) |
|  | 13 | Female, 65 years | No profession |
|  | 14 | Female | Not reported |
|  | 15 | Male | Not reported |
