# Supplementary material for: Satisfaction with care among patients with non-metastatic breast cancer: development and first steps of validation of the REPERES-60 questionnaire
Source: BMC Cancer. 2007 Jul 16;7:129. doi: 10.1186/1471-2407-7-129 (PMC1933545; doi:10.1186/1471-2407-7-129)
Supplement: Additional file 2 — Floor, ceiling effects, missing data, and weighted kappa coefficients of the preliminary items' questionnaire. [file 1471-2407-7-129-S2.doc]

Floor, ceiling effects, missing data, and weighted kappa coefficients of the preliminary items’ questionnaire

| Item | Item wording  *(Italic typeface shows the items that were not retained in the questionnaire)* | One month after initial treatment  (N=820) | | |  | One year later and a week after (N=166) | | Legend of item selection | Number of the final item (REPERES 60) |
| --- | --- | --- | --- | --- | --- | --- | --- | --- | --- |
|  |  | Floor  effect (%) | Ceiling  effect (%) | Missing  data  N (%) |  | Weighted Kappa (n) | 95%  confidence  interval |  |  |
| Q1 | Hours when your doctor’s office is open: GP | 0.4 | 14.3 | 19 ( 2.3) |  | - | - | * | **Item 1** |
| Q2 | Hours when your doctor’s office is open: gynaecologist | 1.2 | 7.2 | 158 (19.3) |  | - | - |  |  |
| Q3 | Hours when your doctor’s is open: radiologist | 0.4 | 8.9 | 42 ( 5.1) |  | - | - |  |  |
| Q4 | Access to care by a surgeon | 1.1 | 14.8 | 37 ( 4.5) |  | - | - | * | **Item 2** |
| Q5 | Access to care by a oncologist | 1.3 | 15.9 | 44 ( 5.4) |  | - | - |  |  |
| Q6 | Access to hospital care | 0.9 | 12.6 | 52 ( 6.3) |  | - | - |  | **Item 3** |
| Q7 | Access to medical care in an emergency | 5.4 | 6.6 | 159 (19.4) |  | - | - |  | **Item 4** |
| Q8 | How easy it is to get an appointment with your GP by phone | 0.9 | 22.0 | 21 ( 2.6) |  | - | - | * | **Item 5** |
| Q9 | How easy it is to get an appointment with your gynaecologist by phone | 3.3 | 10.9 | 148 (18.0) |  | - | - |  |  |
| Q10 | How easy it is to get an appointment with your radiologist by phone | 0.9 | 12.4 | 47 ( 5.7) |  | - | - |  |  |
| Q11 | Length of time you have to wait in your GP’s | 5.2 | 8.2 | 31 ( 3.8) |  | - | - | * | **Item 6** |
| Q12 | Length of time you have to wait in your gynaecologist’s surgery | 5.5 | 4.6 | 152 (18.5) |  | - | - |  |  |
| Q13 | Length of time you have to wait in your radiologist’s | 2.6 | 4.4 | 49 ( 6.0) |  | - | - |  |  |
| Q14 | Length of time you have to wait between the day you make an appointment and the day of consultation with your GP | 0.2 | 21.5 | 28 ( 3.4) |  | - | - | * | **Item 7** |
| Q15 | Length of time you have to wait between the day you make an appointment and the day of consultation with your gynaecologist | 9.6 | 6.0 | 150 (18.3) |  | - | - |  |  |
| Q16 | Length of time you have to wait between the day you make an appointment and the day of consultation with your radiologist | 4.5 | 7.0 | 42 ( 5.1) |  | - | - |  |  |
| Q17 | Availability of medical information or advice over the phone from your GP | 3.2 | 17.8 | 66 ( 8.0) |  | - | - | * | **Item 8** |
| Q18 | Availability of medical information or advice over the phone from your gynaecologist | 6.0 | 7.4 | 218 (26.6) |  | - | - |  |  |
| Q19 | Availability of medical information or advice over the phone from your radiologist | 4.6 | 4.4 | 185 (22.6) |  | - | - |  |  |
| Q20 | How easy it is to see a doctor whenever you need to | 2.0 | 14.9 | 57 ( 7.0) |  | - | - |  | **Item 9** |
| Q21 | Convenience of access to the GP’s | 0.5 | 23.0 | 30 ( 3.7) |  | - | - | * | **Item 10** |
| Q22 | Convenience of access to the gynaecologist’s surgery | 2.1 | 8.7 | 152 (18.5) |  | - | - |  |  |
| Q23 | Convenience of access to the radiologist’s | 1.3 | 11.7 | 50 ( 6.1) |  | - | - |  |  |
| Q24 | The care taken by the GP you know in examining you and the accuracy of their diagnoses | 2.3 | 18.7 | 69 ( 8.4) |  | - | - | * | **Item 11** |
| Q25 | The care taken by the gynaecologist you know in examining you and the accuracy of their diagnoses | 4.1 | 16.8 | 165 (20.1) |  | - | - |  |  |
| Q26 | The care taken by the radiologist you know in examining you and the accuracy of their diagnoses | 3.3 | 25.2 | 29 ( 3.5) |  | - | - |  |  |
| Q27 | Skill and experience of the GP you know | 1.3 | 20.4 | 58 ( 7.1) |  | - | - | * | **Item 12** |
| Q28 | Skill and experience of the gynaecologist you know | 2.3 | 17.2 | 166 (20.2) |  | - | - |  |  |
| Q29 | Skill and experience of the radiologist you know | 2.2 | 23.8 | 44 ( 5.4) |  | - | - |  |  |

* Rearrange in one-single item

** Deleted: missing data

*** Deleted: redundancy

**** Deleted: Items included in the principal component analysis and not included in the final questionnaire

| Item | Item wording  *(Italic typeface shows the items that were not retained in the questionnaire)* | One month after initial treatment  (N=820) | | |  | One year later and a week after (N=166) | | Legend of item selection | Number of the final item (REPERES 60) |
| --- | --- | --- | --- | --- | --- | --- | --- | --- | --- |
|  |  | Floor  effect (%) | Ceiling  effect (%) | Missing  data  N (%) |  | Weighted Kappa (n) | 95%  confidence  interval |  |  |
| Q30 | Thoroughness of GP you know in choosing treatment | 1.5 | 28.4 | 44 ( 5.4) |  | - | - | * | **Item 13** |
| Q31 | Thoroughness of gynaecologist you know in choosing treatment | 3.7 | 17.3 | 196 (23.9) |  | - | - |  |  |
| Q32 | Thoroughness of radiologist you know in choosing treatment | 2.0 | 18.5 | 132 (16.1) |  | - | - |  |  |
| Q33 | The care taken by the surgeons you know in examining you and the accuracy of their diagnoses | 1.0 | 37.1 | 18 ( 2.2) |  | - | - | * | **Item 14** |
| Q34 | The care taken by the oncologists you know in examining you and the accuracy of their diagnoses | 0.1 | 36.8 | 36 ( 4.4) |  | - | - |  |  |
| Q35 | Competence and experience of the surgeons you know | 0.2 | 38.4 | 25 ( 3.0) |  | - | - | * | **Item 15** |
| Q36 | Competence and experience of the oncologists you know | 0.1 | 35.6 | 42 ( 5.1) |  | - | - |  |  |
| Q37 | Thoroughness of the surgeons you know in choosing treatment | 0.6 | 33.4 | 38 ( 4.6) |  | - | - | * | **Item 16** |
| Q38 | Thoroughness of the oncologists you know in choosing treatment | 0.2 | 34.6 | 30 ( 3.7) |  | - | - |  |  |
| Q39 | The explanations provided by the GPs you know on medical procedures and tests | 1.3 | 20.5 | 42 ( 5.1) |  | - | - | * | **Item 17** |
| Q40 | The explanations provided by the gynaecologists you know on medical procedures and tests | 3.2 | 12.8 | 179 (21.8) |  | - | - |  |  |
| Q41 | The explanations provided by the radiologists you know on medical procedures and tests | 2.3 | 14.3 | 90 (11.0) |  | - | - |  |  |
| Q42 | The attention paid by GPs you know to what you say | 0.7 | 24.0 | 35 ( 4.3) |  | - | - | * | **Item 18** |
| Q43 | The attention paid by gynaecologists you know to what you say | 2.7 | 13.3 | 186 (22.7) |  | - | - |  |  |
| Q44 | The attention paid by radiologists you know to what you say | 2.0 | 13.9 | 118 (14.4) |  | - | - |  |  |
| Q45 | Advice on preventative measures you receive from GPs to stay healthy | 2.3 | 17.7 | 57 ( 7.0) |  | - | - | * | **Item 19** |
| Q46 | Advice on preventative measures you receive from gynaecologists to stay healthy | 4.1 | 9.1 | 222 (27.1) |  | - | - |  |  |
| Q47 | Advice on preventative measures you receive from radiologists to stay healthy | 4.0 | 9.9 | 187 (22.8) |  | - | - |  |  |
| Q48 | The explanations provided by the surgeons you know on medical procedures and tests | 2.4 | 26.5 | 21 ( 2.6) |  | - | - | * | **Item 20** |
| Q49 | The explanations provided by the oncologists you know on medical procedures and tests | 1.6 | 25.7 | 32 ( 3.9) |  | - | - |  |  |
| Q50 | The attention paid by surgeons you know to what you say | 2.3 | 22.6 | 31 ( 3.8) |  | - | - | * | **Item 21** |
| Q51 | The attention paid by oncologists you know to what you say | 1.8 | 24.3 | 39 ( 4.8) |  | - | - |  |  |
| Q52 | Advice on preventative measures you receive from surgeons to stay healthy | 2.9 | 17.2 | 61 ( 7.4) |  | - | - | * | **Item 22** |
| Q53 | Advice on preventative measures you receive from oncologists to stay healthy | 2.4 | 18.9 | 59 ( 7.2) |  | - | - |  |  |
| Q54 | The number of GPs you can consult | 1.1 | 15.0 | 25 ( 3.0) |  | - | - | * | **Item 23** |
| Q55 | The number of gynaecologists you can consult | 8.4 | 6.3 | 109 (13.3) |  | - | - |  |  |
| Q56 | The number of radiologists you can consult | 4.3 | 8.8 | 42 ( 5.1) |  | - | - |  |  |
| Q57 | How easy it is to consult the GP of you choice | 0.9 | 19.6 | 25 ( 3.0) |  | - | - | * | **Item 24** |
| Q58 | How easy it is to consult the gynaecologist of you choice | 4.6 | 11.5 | 117 (14.3) |  | - | - |  |  |
| Q59 | How easy it is to consult the radiologist of you choice | 2.7 | 13.8 | 47 ( 5.7) |  | - | - |  |  |
| Q60 | The number of surgeons you can consult | 6.7 | 8.0 | 51 ( 6.2) |  | - | - | * | **Item 25** |
| Q61 | The number of oncologists you can consult | 10.1 | 7.8 | 59 ( 7.2) |  | - | - |  |  |
| Q62 | How easy it is to consult the surgeon of your choice | 4.8 | 10.4 | 62 ( 7.6) |  | - | - | * | **Item 26** |
| Q63 | How easy it is to consult the oncologist of your choice | 7.4 | 10.0 | 72 ( 8.8) |  | - | - |  |  |

* Rearrange in one-single item

** Deleted: missing data

*** Deleted: redundancy

**** Deleted: Items included in the principal component analysis and not included in the final questionnaire

| Item | Item wording  *(Italic typeface shows the items that were not retained in the questionnaire)* | One month after initial treatment  (N=820) | | |  | One year later and a week after (N=166) | | Legend of item selection | Number of the final item (REPERES 60) |
| --- | --- | --- | --- | --- | --- | --- | --- | --- | --- |
|  |  | Floor  effect (%) | Ceiling  effect (%) | Missing  data  N (%) |  | Weighted Kappa (n) | 95%  confidence  interval |  |  |
| Q64 | Kindness (friendliness) and courtesy of doctors | 0.1 | 24.5 | 5 ( 0.6) |  | 0.68 (161) | 0.58-0.77 |  | **Item 27** |
| Q65 | Interest taken by doctors in you and your health problems | 0.5 | 20.6 | 10 ( 1.2) |  | 0.66 (159) | 0.57-0.75 |  | **Item 28** |
| Q66 | Respect shown to you by doctors and attention to privacy | 0.4 | 23.0 | 8 ( 1.0) |  | 0.67 (160) | 0.57-0.77 |  | **Item 29** |
| Q67 | The ability of doctors to reassure you and give you support | 3.8 | 11.2 | 11 ( 1.3) |  | 0.58 (158) | 0.45-0.71 |  | **Item 30** |
| Q68 | I am very satisfied with the care I receive | 0.2 | 56.1 | 10 ( 1.2) |  | 0.55 (158) | 0.32-0.77 |  | **Item 31** |
| Q69 | Some things in the care I receive could be better | 20.1 | 6.6 | 36 ( 4.4) |  | 0.44 (154) | 0.27-0.61 |  | **Item 32** |
| Q70 | The care I receive is practically perfect | 0.5 | 36.1 | 22 ( 2.7) |  | 0.62 (158) | 0.45-0.79 |  | **Item 33** |
| Q71 | I am dissatisfied with some things in the care I receive | 6.6 | 42.3 | 54 ( 6.6) |  | 0.60 (148) | 0.48-0.73 |  | **Item 34** |
| Q72 | The ability of your health cover to compensate for medical expenses and loss of income | 5.1 | 19.9 | 44 ( 5.4) |  | 0.75 (156) | 0.67-0.84 |  | **Item 35** |
| Q73 | The range of costs that are reimbursed | 2.1 | 19.8 | 48 ( 5.9) |  | 0.75 (151) | 0.66-0.83 |  | **Item 36** |
| Q74 | The reimbursement of your consultation fees | 1.3 | 27.2 | 33 ( 4.0) |  | 0.68 (159) | 0.57-0.80 |  | **Item 37** |
| Q75 | The reimbursement of your hospital expenses | 2.1 | 28.5 | 38 ( 4.6) |  | 0.76 (157) | 0.68-0.84 |  | **Item 38** |
| Q76 | The reimbursement of your expenditure for medication | 1.2 | 27.2 | 33 ( 4.0) |  | 0.78 (161) | 0.71-0.85 |  | **Item 39** |
| Q77 | The time required to obtain reimbursement of medical expenses incurred | 4.3 | 11.8 | 67 ( 8.2) |  | 0.72 (148) | 0.62-0.82 | - |  |
| *Q78* | *The amounts reimbursed for non-medical expenses (wig, prosthesis, special underwear)* | *23.2* | *3.7* | *313 (38.2)* |  | *0.87 ( 81)* | *0.82-0.93* | **** |  |
| *Q79* | *The amounts reimbursed for expenses relating to help you needed after discharge (home help, psychologist, child-minder)* | *15.1* | *3.2* | *438 (53.4)* |  | *0.82 ( 66)* | *0.71-0.93* | **** |  |
| *Q80* | *The explanations given you to help you understand your treatment better, and to help you to prepare for it* | *2.7* | *11.8* | *16 ( 2.0)* |  | *0.71 (162)* | *0.62-0.79* | ***** |  |
| Q81 | The explanations given you to help you prepare for the consequences of surgery | 3.0 | 11.2 | 32 ( 3.9) |  | 0.66 (158) | 0.54-0.78 |  | **Item 40** |
| Q82 | The information given you on your treatment as a whole | 1.5 | 11.5 | 17 ( 2.1) |  | 0.75 (162) | 0.68-0.82 |  | **Item 41** |
| Q83 | The information given you on the consequences of the illness | 6.2 | 9.5 | 24 ( 2.9) |  | 0.71 (158) | 0.59-0.83 |  | **Item 42** |
| Q84 | The information given you on side effects of the treatment | 8.0 | 9.4 | 27 ( 3.3) |  | 0.71 (155) | 0.62-0.80 |  | **Item 43** |
| Q85 | The information given you on pain management | 6.6 | 7.2 | 77 ( 9.4) |  | 0.77 (143) | 0.69-0.85 |  | **Item 44** |
| Q86 | The information given you on the possibilities for breast reconstruction | 9.1 | 5.1 | 372 (45.4) |  | 0.73 ( 90) | 0.60-0.86 |  | **Item 45** |
| *Q87* | *The care taken by doctors to consult you on how much information to give to your family or those close to you* | *12.2* | *5.2* | *151 (18.4)* |  | *0.75 (133)* | *0.64-0.85* | **** |  |
| *Q88* | *How far the doctors abided by your opinion on how much information to give to your family/those close* | *7.4* | *6.6* | *158 (19.3)* |  | *0.61 (126)* | *0.44-0.77* | **** |  |
| Q89 | The quality of the information received about your disease | 3.8 | 8.4 | 47 ( 5.7) |  | 0.67 (157) | 0.56-0.77 |  | **Item 46** |
| *Q90* | *The information given you on the possibility of consulting your medical file* | *22.0* | *4.0* | *164 (20.0)* |  | *0.76 (130)* | *0.65-0.86* | **** |  |
| *Q91* | *How easy it was to have access to your medical file* | *19.6* | *3.7* | *208 (25.4)* |  | *0.83 (125)* | *0.77-0.89* | **** |  |
| *Q92* | *The information given you about patient groups that might help you* | *20.7* | *3.0* | *205 (25.0)* |  | *0.72 (117)* | *0.61-0.82* | **** |  |
| *Q93* | *The efficiency of the care you received in the hospital and/or clinic* | *0.2* | *21.3* | *9 ( 1.1)* |  | *0.73 (164)* | *0.63-0.83* | ****** |  |
| *Q94* | *The efficiency of any care you may have received in your home* | *1.0* | *13.2* | *236 (28.8)* |  | *0.78 (114)* | *0.68-0.88* | ****** |  |

* Rearrange in one-single item

** Deleted: missing data

*** Deleted: redundancy

**** Deleted: Items included in the principal component analysis and not included in the final questionnaire

| Item | Item wording  *(Italic typeface shows the items that were not retained in the questionnaire)* | One month after initial treatment  (N=820) | | |  | One year later and a week after (N=166) | | | Legend of item selection | Number of the final item (REPERES 60) |
| --- | --- | --- | --- | --- | --- | --- | --- | --- | --- | --- |
|  |  | Floor  effect (%) | Ceiling  effect (%) | Missing  data  N (%) |  | Weighted Kappa (n) | | 95%  confidence  interval |  |  |
| *Q95* | *The efficiency of pain management medication* | *3.9* | *11.7* | *133 (16.2)* |  | | *0.73 (126)* | *0.61-0.84* | ****** |  |
| *Q96* | *The efficiency of treatment given you to cater for side effects of the disease and/or the treatment* | *6.7* | *7.3* | *116 (14.1)* |  | *0.71 (132)* | | *0.58-0.83* | ****** |  |
| *Q97* | *How the doctors reacted if you asked for a different mode of treatment* | *2.9* | *3.2* | *334 (40.7)* |  | *0.42 ( 96)* | | *0.20-0.64* | **** |  |
| *Q98* | *The way your treatment was discussed among the different doctors* | *1.6* | *10.9* | *161 (19.6)* |  | *0.72 (123)* | | *0.61-0.82* | **** |  |
| *Q99* | *Your degree of participation in the choice of the treatment* | *9.5* | *5.0* | *219 (26.7)* |  | *0.72 (109)* | | *0.61-0.83* | **** |  |
| *Q100* | *The information you were given on the organisation and follow-up of the care provided for you* | *4.0* | *9.9* | *97 (11.8)* |  | *0.50 (131)* | | *0.34-0.66* | **** |  |
| *Q101* | *The possibility of being catered for by a single medical team* | *4.3* | *9.5* | *174 (21.2)* |  | *0.47 (121)* | | *0.27-0.66* | **** |  |
| Q102 | Communication overall among all the different doctors who have cared for you since your diagnosis | 3.3 | 15.2 | 89 (10.9) |  | 0.58 (137) | | 0.41-0.74 |  | **Item 47** |
| *Q103* | *Communication between the radiologists on the one hand, and surgeons and oncologists on the other* | *2.77* | *15.1* | *97 (11.8)* |  | *0.52 (138)* | | *0.35-0.69* | **** |  |
| Q104 | Time-lapse before receiving surgery (if applicable) | 1.5 | 16.1 | 113 (13.8) |  | 0.68 (130) | | 0.58-0.79 |  | **Item 48** |
| Q105 | Time-lapse before receiving chemotherapy (if applicable) | 0.4 | 10.7 | 383 (46.7) |  | 0.69 ( 64) | | 0.55-0.82 |  | **Item 49** |
| Q106 | Time-lapse before receiving radiotherapy (if applicable) | 1.7 | 15.1 | 50 ( 6.1) |  | 0.72 (146) | | 0.61-0.83 |  | **Item 50** |
| Q107 | Time taken by the specialist doctors to inform your GP | 2.6 | 15.1 | 72 ( 8.8) |  | 0.59 (150) | | 0.43-0.74 |  | **Item 51** |
| *Q108* | *Time taken by doctors to exchange information on the way in which you are to be cared for* | *2.2* | *11.1* | *162 (19.8)* |  | *0.55 (127)* | | *0.38-0.72* | **** |  |
| Q109 | In case of hospitalisation, the opportunities to talk to someone about your health problems when you needed to (people close to you, an association, medical and non-medical staff) | 5.9 | 9.4 | 103 (12.6) |  | 0.58 (140) | | 0.45-0.70 |  | **Item 52** |
| Q110 | When at home, the opportunities to talk to someone about your health problems when you needed to (people close to you, an association, medical and non-medical staff) | 7.9 | 9.0 | 131 (16.0) |  | 0.65 (135) | | 0.53-0.76 |  | **Item 53** |
| Q111 | The psychological support that the doctors provided you with | 4.9 | 9.1 | 92 (11.2) |  | 0.62 (138) | | 0.48-0.76 |  | **Item 54** |
| Q112 | The psychological support that the nurses provided you with | 2.6 | 12.2 | 97 (11.8) |  | 0.60 (136) | | 0.46-0.74 |  | **Item 55** |
| *Q113* | *The information given you on assistance you are allowed once you return home* | *28.4* | *3.4* | *192 (23.4)* |  | *0.65 (115)* | | *0.51-0.79* | **** |  |
| *Q114* | *Preparation for your return home with the care team (doctors, nurses, care assistants etc)* | *14.1* | *5.4* | *206 (25.1)* |  | *0.63 (116)* | | *0.51-0.76* | **** |  |
| *Q115* | *The quality of the care received when you returned home* | *14.6* | *4.0* | *321 (39.1)* |  | *0.67 ( 95)* | | *0.54-0.79* | **** |  |
| Q116 | The assistance provided by the medical world throughout your care | 2.1 | 12.4 | 106 (12.9) |  | 0.63 (142) | | 0.49-0.76 |  | **Item 56** |
| *Q117* | *The assistance provided by associations and informal groups throughout your care* | *14.1* | *2.0* | *459 (56.0)* |  | *0.73 ( 73)* | | *0.60-0.87* | **** |  |
| Q118 | The quality of the consultation premises you visited (quietness, atmosphere etc)) | 2.8 | 11.1 | 16 ( 2.0) |  | 0.62 (155) | | 0.51-0.72 |  | **Item 57** |
| Q119 | The quality of the hospital wards you have been in (quietness, atmosphere etc) | 1.2 | 13.2 | 24 ( 2.9) |  | 0.66 (155) | | 0.54-0.77 |  | **Item 58** |
| Q120 | The respect for your privacy in the consultation premises you visited | 1.6 | 14.9 | 25 ( 3.0) |  | 0.75 (152) | | 0.66-0.84 |  | **Item 59** |
| Q121 | The respect for you privacy on hospital wards that you have been in | 1.2 | 15.4 | 36 ( 4.4) |  | 0.67 (150) | | 0.52-0.81 |  | **Item 60** |

* Rearrange in one-single item

** Deleted: missing data

*** Deleted: redundancy

**** Deleted: Items included in the principal component analysis and not included in the final questionnaire
