# Supplementary material for: Satisfaction with care among patients with non-metastatic breast cancer: development and first steps of validation of the REPERES-60 questionnaire
Source: BMC Cancer. 2007 Jul 16;7:129. doi: 10.1186/1471-2407-7-129 (PMC1933545; doi:10.1186/1471-2407-7-129)
Supplement: Additional file 3 — Principal components factor analysis (varimax rotation) computed with the 60 items of the final questionnaire (n = 174). [file 1471-2407-7-129-S3.doc]

Principal components factor analysis (varimax rotation) computed with the 60 items of the final questionnaire (n=174)

| Item | Label | Factor 1 | Factor 2 | Factor 3 | Factor 4 | Factor 5 | Factor 6 | Factor 7 | Factor 8 | Factor 9 | Factor 10 | Factor 11 | Factor 12 |
| --- | --- | --- | --- | --- | --- | --- | --- | --- | --- | --- | --- | --- | --- |
| I1 | Hours when your doctor’s office (GP and/or gynaecologist) is open | 0.40 | 0.10 | 0.18 | 0.14 | -0.13 | **0.51** | 0.08 | 0.16 | 0.03 | 0.00 | 0.28 | 0.30 |
| I2 | How easy it is to get an appointment with your GP and/or gynaecologist by phone | 0.40 | 0.20 | 0.13 | 0.23 | -0.04 | **0.62** | 0.12 | 0.16 | -0.03 | 0.08 | 0.25 | 0.10 |
| I3 | Length of time you have to wait in your GP’s and/or gynaecologist’s surgery | 0.12 | 0.10 | -0.03 | 0.04 | 0.24 | **0.70** | -0.05 | 0.18 | 0.19 | 0.04 | 0.04 | 0.11 |
| I4 | Length of time you have to wait between the day you make an appointment and the day of consultation with your GP and/or gynaecologist | 0.38 | 0.14 | 0.12 | 0.06 | 0.01 | **0.65** | 0.20 | 0.17 | 0.13 | 0.05 | 0.10 | 0.09 |
| I5 | Availability of medical information or advice over the phone from your GP and/or gynaecologist | **0.49** | 0.20 | 0.07 | 0.04 | 0.15 | **0.49** | 0.13 | -0.09 | 0.18 | 0.02 | 0.12 | 0.11 |
| I6 | How easy it is to see a doctor whenever you need to (GP and/or gynaecologist) | 0.27 | 0.14 | 0.13 | 0.07 | 0.02 | **0.72** | 0.11 | 0.27 | 0.06 | 0.08 | 0.06 | 0.04 |
| I7 | Convenience of access to the GP's and/or gynaecologist’s surgery | 0.37 | 0.23 | 0.17 | 0.30 | 0.01 | **0.47** | 0.10 | 0.06 | 0.05 | 0.13 | 0.02 | 0.00 |
| I8 | Access to care by a specialist (oncologist, surgeon) | 0.26 | 0.28 | 0.04 | 0.35 | 0.16 | 0.22 | 0.22 | 0.19 | 0.20 | 0.00 | 0.15 | **0.49** |
| I9 | Access to hospital care | 0.22 | 0.19 | 0.14 | 0.25 | 0.10 | 0.12 | 0.26 | 0.29 | 0.17 | 0.11 | 0.14 | **0.60** |
| I10 | Access to medical care in an emergency | 0.17 | 0.08 | 0.16 | 0.02 | 0.14 | 0.16 | -0.08 | 0.16 | 0.05 | 0.15 | 0.08 | **0.75** |
| I11 | The care taken by the GPs and/or gynaecologists you know in examining you and the accuracy of their diagnoses | **0.72** | 0.13 | 0.17 | 0.02 | 0.07 | 0.11 | 0.18 | 0.22 | 0.12 | 0.01 | 0.00 | 0.33 |
| I12 | Skill and experience of the GPs and/or gynaecologists you know | **0.78** | 0.07 | 0.15 | 0.12 | 0.06 | 0.19 | 0.12 | 0.16 | 0.08 | 0.02 | 0.03 | 0.21 |
| I13 | Thoroughness of GPs and/or gynaecologists you know in choosing treatment | **0.83** | 0.13 | 0.10 | 0.21 | 0.08 | 0.17 | 0.16 | 0.10 | 0.07 | -0.04 | 0.05 | 0.06 |
| I14 | The explanations provided by the GPs and/or gynaecologists you know on medical procedures and tests | **0.76** | 0.26 | 0.06 | 0.19 | 0.15 | 0.23 | -0.05 | 0.04 | 0.14 | 0.05 | 0.16 | -0.05 |
| I15 | The attention paid by GPs and/or gynaecologists you know to what you say | **0.78** | 0.16 | 0.09 | 0.17 | 0.19 | 0.19 | 0.08 | 0.09 | 0.12 | 0.13 | 0.14 | -0.01 |
| I16 | Advice on preventative measures you receive from GPs and/or gynaecologists to stay healthy | **0.68** | 0.20 | 0.04 | 0.13 | 0.29 | 0.22 | 0.06 | 0.09 | 0.08 | 0.15 | 0.04 | 0.00 |
| I17 | The care taken by the (medical) specialists (oncologists, surgeons) you know in examining you and the accuracy of their diagnoses | 0.24 | 0.18 | 0.21 | **0.72** | 0.17 | 0.08 | 0.15 | 0.09 | 0.27 | 0.10 | 0.08 | 0.10 |
| I18 | Competence and experience of the specialists (oncologists, surgeons) you know | 0.22 | 0.07 | 0.12 | **0.75** | 0.21 | 0.06 | 0.17 | 0.11 | 0.25 | 0.19 | 0.04 | 0.10 |
| I19 | Thoroughness of the specialists (oncologists, surgeons) you know in choosing treatment | 0.20 | 0.13 | 0.11 | **0.73** | 0.17 | 0.14 | 0.18 | 0.15 | 0.27 | 0.17 | 0.12 | 0.03 |
| I20 | The explanations provided by the specialists (oncologists, surgeons) you know on medical procedures and tests | 0.13 | **0.49** | 0.13 | **0.51** | 0.39 | 0.22 | 0.04 | 0.07 | 0.03 | 0.06 | -0.01 | 0.11 |
| I21 | The attention paid by specialists (oncologists, surgeons) you know to what you say | 0.18 | 0.34 | 0.11 | **0.54** | **0.49** | 0.23 | 0.11 | -0.03 | -0.01 | 0.13 | 0.10 | 0.06 |
| I22 | Advice on preventative measures you receive from specialists (oncologists, surgeons) to stay healthy | 0.20 | 0.40 | 0.06 | 0.39 | **0.53** | 0.19 | 0.10 | -0.02 | 0.04 | 0.10 | 0.07 | 0.15 |
| I23 | The number of GPs and/or gynaecologists you can consult | 0.41 | 0.34 | 0.07 | 0.05 | 0.03 | 0.20 | 0.08 | **0.61** | -0.10 | 0.14 | 0.10 | 0.06 |
| I24 | How easy it is to consult the GP and/or the gynaecologist of you choice | 0.33 | 0.18 | 0.09 | 0.16 | 0.02 | 0.26 | 0.07 | **0.69** | 0.08 | 0.10 | 0.08 | 0.00 |
| I25 | The number of specialists (oncologists, surgeons) you can consult | 0.06 | 0.08 | 0.11 | 0.05 | 0.17 | 0.16 | 0.08 | **0.81** | 0.06 | 0.07 | 0.06 | 0.16 |
| I26 | How easy it is to consult the specialist (oncologist, surgeon) of your choice | 0.07 | 0.10 | 0.12 | 0.11 | 0.28 | 0.18 | 0.05 | **0.74** | 0.18 | -0.03 | 0.16 | 0.21 |
| I27 | Kindness (friendliness) and courtesy of doctors | 0.28 | 0.18 | 0.22 | 0.34 | **0.56** | 0.00 | 0.18 | 0.28 | 0.12 | 0.23 | 0.11 | 0.07 |
| I28 | Interest taken by doctors in you and your health problems | 0.18 | 0.28 | 0.18 | 0.20 | **0.70** | 0.05 | 0.14 | 0.24 | 0.13 | 0.24 | 0.08 | 0.14 |
| I29 | Respect shown to you by doctors and attention to privacy | 0.21 | 0.15 | 0.22 | 0.32 | **0.62** | -0.01 | 0.19 | 0.30 | 0.08 | 0.21 | 0.12 | 0.06 |
| I30 | The ability of doctors to reassure you and give you support | 0.11 | 0.28 | 0.08 | 0.16 | **0.72** | 0.03 | 0.16 | 0.08 | 0.11 | 0.27 | 0.13 | 0.08 |

Bold typeface shows the component upon which each item load most highly

Factor 1: Competence and attention paid by doctors before your treatment Factor 5 : Human qualities shown by doctors Factor 9 : Organisation and follow-up of medical care

Factor 2: Listening abilities and information provided by doctors Factor 6 : Access to primary care Factor 10 : Satisfaction overall

Factor 3: Cover for medical expenses Factor 7 : Material environment Factor 11 : Organisation and follow-up of medical care

Factor 4: Competence and attention paid by doctors for your treatment Factor 8 : Choice among different doctors Factor 12 : Access to secondary care

| Item | Label | Factor 1 | Factor 2 | Factor 3 | Factor 4 | Factor 5 | Factor 6 | Factor 7 | Factor 8 | Factor 9 | Factor 10 | Factor 11 | Factor 12 |
| --- | --- | --- | --- | --- | --- | --- | --- | --- | --- | --- | --- | --- | --- |
| I31 | I am very satisfied with the care I receive | 0.24 | 0.23 | 0.02 | 0.09 | 0.27 | 0.00 | 0.14 | 0.11 | 0.20 | **0.64** | -0.03 | 0.06 |
| I32 | Some things in the care I receive could be better | -0.11 | 0.11 | 0.11 | 0.10 | 0.08 | 0.04 | 0.13 | -0.04 | 0.13 | **0.78** | 0.11 | 0.09 |
| I33 | The care I receive is practically perfect | 0.17 | 0.14 | 0.11 | 0.09 | 0.13 | 0.01 | 0.19 | 0.21 | 0.02 | **0.70** | 0.06 | 0.01 |
| I34 | I am dissatisfied with some things in the care I receive | 0.03 | 0.10 | 0.00 | 0.15 | 0.19 | 0.15 | 0.00 | 0.00 | 0.04 | **0.78** | 0.10 | 0.06 |
| I35 | The ability of your health cover to compensate for medical expenses and loss of income | 0.06 | 0.09 | **0.75** | 0.02 | 0.13 | 0.01 | -0.03 | -0.01 | 0.06 | 0.09 | 0.16 | 0.09 |
| I36 | The range of costs that are reimbursed | 0.10 | 0.17 | **0.90** | 0.03 | 0.11 | 0.12 | 0.08 | 0.15 | 0.14 | 0.05 | -0.02 | 0.05 |
| I37 | The reimbursement of your consultation fees | 0.10 | 0.17 | **0.90** | 0.03 | 0.11 | 0.12 | 0.08 | 0.15 | 0.14 | 0.05 | -0.02 | 0.05 |
| I38 | The reimbursement of your hospital expenses | 0.11 | 0.06 | **0.78** | 0.16 | 0.00 | 0.01 | 0.20 | 0.02 | 0.12 | -0.01 | 0.09 | 0.12 |
| I39 | The reimbursement of your expenditure for medication | 0.14 | 0.08 | **0.76** | 0.24 | 0.04 | 0.12 | 0.05 | 0.05 | 0.08 | 0.06 | 0.19 | -0.03 |
| I40 | The explanations given you to help you prepare for the consequences of surgery | 0.19 | **0.64** | 0.06 | 0.37 | 0.02 | 0.18 | 0.15 | 0.21 | 0.10 | 0.02 | 0.05 | -0.03 |
| I41 | The information given you on your treatment as a whole | 0.27 | **0.70** | 0.07 | 0.20 | 0.14 | 0.16 | 0.20 | 0.12 | 0.18 | 0.17 | 0.05 | -0.02 |
| I42 | The information given you on the consequences of the illness | 0.16 | **0.77** | 0.10 | 0.03 | 0.22 | 0.12 | 0.01 | 0.15 | 0.18 | 0.13 | 0.13 | 0.07 |
| I43 | The information given you on side effects of the treatment | 0.10 | **0.80** | 0.12 | 0.06 | 0.11 | 0.10 | 0.15 | 0.05 | 0.15 | 0.17 | 0.10 | 0.12 |
| I44 | The information given you on pain management | 0.12 | **0.69** | 0.20 | 0.01 | 0.25 | 0.12 | 0.19 | 0.07 | 0.15 | 0.10 | 0.12 | 0.19 |
| I45 | The information given you on the possibilities for breast reconstruction | 0.15 | **0.60** | 0.19 | 0.21 | 0.00 | 0.06 | 0.10 | 0.11 | 0.14 | 0.05 | 0.20 | 0.06 |
| I46 | The quality of the information received about your disease | 0.22 | **0.60** | 0.19 | 0.16 | 0.31 | 0.10 | 0.06 | 0.08 | 0.17 | 0.11 | 0.23 | -0.03 |
| I47 | Communication overall among all the different doctors who have cared for you since your diagnosis | 0.23 | 0.35 | 0.07 | 0.14 | 0.30 | 0.05 | 0.29 | 0.16 | **0.45** | 0.16 | 0.23 | -0.07 |
| I48 | Time-lapse before receiving surgery (if applicable) | 0.18 | 0.25 | 0.12 | 0.30 | 0.06 | 0.05 | 0.12 | 0.13 | **0.75** | 0.15 | 0.08 | 0.11 |
| I49 | Time-lapse before receiving chemotherapy (if applicable) | 0.15 | 0.26 | 0.20 | 0.26 | 0.09 | 0.09 | 0.12 | 0.01 | **0.76** | 0.09 | 0.14 | 0.05 |
| I50 | Time-lapse before receiving radiotherapy (if applicable) | 0.08 | 0.16 | 0.26 | 0.14 | 0.09 | 0.18 | 0.09 | 0.03 | **0.73** | 0.07 | 0.08 | 0.10 |
| I51 | Time taken by the specialist doctors to inform your GP | 0.22 | 0.33 | 0.10 | 0.03 | 0.13 | 0.16 | 0.17 | 0.15 | 0.44 | 0.14 | 0.29 | -0.02 |
| I52 | In case of hospitalisation, the opportunities to talk to someone about your health problems when you needed to | 0.13 | 0.29 | 0.17 | 0.14 | 0.08 | 0.21 | 0.17 | 0.14 | 0.12 | 0.10 | **0.68** | 0.16 |
| I53 | When at home, the opportunities to talk to someone about your health problems when you needed to | 0.20 | 0.22 | 0.23 | 0.12 | 0.13 | 0.11 | 0.12 | 0.17 | 0.18 | 0.07 | **0.66** | 0.16 |
| I54 | The psychological support that the doctors provided you with | 0.19 | **0.50** | 0.07 | 0.00 | 0.42 | 0.17 | 0.17 | 0.10 | 0.21 | 0.16 | 0.38 | 0.08 |
| I55 | The psychological support that the nurses provided you with | -0.04 | 0.21 | 0.15 | 0.11 | 0.23 | 0.23 | 0.34 | 0.07 | 0.13 | 0.22 | **0.46** | -0.06 |
| I56 | The assistance provided by the medical world throughout your care | 0.15 | 0.32 | 0.03 | -0.05 | 0.36 | 0.08 | 0.33 | 0.14 | 0.27 | 0.24 | **0.49** | -0.03 |
| I57 | The quality of the consultation premises you visited (quietness, atmosphere etc)) | 0.12 | 0.09 | 0.05 | -0.02 | 0.32 | 0.13 | **0.76** | 0.09 | 0.05 | 0.12 | -0.03 | 0.13 |
| I58 | The quality of the hospital wards you have been in (quietness, atmosphere etc) | 0.10 | 0.22 | 0.10 | 0.09 | 0.06 | 0.05 | **0.77** | 0.08 | 0.13 | 0.07 | 0.07 | 0.06 |
| I59 | The respect for your privacy in the consultation premises you visited | 0.17 | 0.13 | 0.11 | 0.33 | 0.11 | 0.11 | **0.67** | 0.03 | 0.10 | 0.18 | 0.26 | -0.04 |
| I60 | The respect for you privacy on hospital wards that you have been in | 0.15 | 0.14 | 0.14 | 0.38 | -0.01 | 0.07 | **0.68** | 0.03 | 0.12 | 0.09 | 0.31 | -0.06 |

Bold typeface shows the component upon which each item load most highly

Factor 1: Competence and attention paid by doctors before your treatment Factor 5 : Human qualities shown by doctors Factor 9 : Organisation and follow-up of medical care

Factor 2: Listening abilities and information provided by doctors Factor 6 : Access to primary care Factor 10 : Satisfaction overall

Factor 3: Cover for medical expenses Factor 7 : Material environment Factor 11 : Organisation and follow-up of medical care

Factor 4: Competence and attention paid by doctors for your treatment Factor 8 : Choice among different doctors Factor 12 : Access to secondary care
