# Supplementary material for: Satisfaction with care among patients with non-metastatic breast cancer: development and first steps of validation of the REPERES-60 questionnaire
Source: BMC Cancer. 2007 Jul 16;7:129. doi: 10.1186/1471-2407-7-129 (PMC1933545; doi:10.1186/1471-2407-7-129)
Supplement: Additional file 4 — Results of psychometric tests for the final questionnaire REPERES-60. [file 1471-2407-7-129-S4.doc]

Results of psychometric tests for the final questionnaire REPERES-60

|  | **39 items** | | | | | | | | |  | **21 new items** | | | | |
| --- | --- | --- | --- | --- | --- | --- | --- | --- | --- | --- | --- | --- | --- | --- | --- |
|  | **ASP** | **ASS** | **COMG** | **COMPS** | **COMMS** | **PC** | **QH** | **SG** | **CM** |  | **INF** | **ORG** | **PSY** | **ENV** |  |
| **Intraclass correlation coefficient** | 0.85 | 0.62 | 0.82 | 0.66 | 0.71 | 0.80 | 0.75 | 0.72 | 0.82 |  | 0.82 | 0.72 | 0.74 | 0.75 |  |
| **Number of items** | 7 | 3 | 6 | 3 | 3 | 4 | 4 | 4 | 5 |  | 7 | 5 | 5 | 4 |  |
| **% completed items^a^** | 85.5 | 75.9 | 91.5 | 97.6 | 94.6 | 91.2 | 97.8 | 91.6 | 87.9 |  | 52.8 | 44.0 | 72.9 | 94.9 |  |
| **% computable items^b^** | 98.8 | 97.0 | 99.0 | 99.0 | 99.0 | 97.8 | 99.2 | 97.8 | 97.2 |  | 97.1 | 93.5 | 89.6 | 98.1 |  |
|  |  |  |  |  |  |  |  |  |  |  |  |  |  |  |  |
| **Range of convergent item-scale correlations^c^** | 0.56–0.74 | 0.56–0.73 | 0.74–0.83 | 0.85–0.90 | 0.79–0.86 | 0.69–0.74 | 0.82–0.89 | 0.51–0.57 | 0.68–0.83 |  | 0.64–0.87 | 0.64–0.76 | 0.67–0.78 | 0.73–0.80 |  |
| **Reliability^d^** | 0.88 | 0.82 | 0.93 | 0.94 | 0.92 | 0.87 | 0.94 | 0.74 | 0.90 |  | 0.93 | 0.87 | 0.88 | 0.89 |  |
|  |  |  |  |  |  |  |  |  |  |  |  |  |  |  |  |
| **Mean score** | 60.5 | 59.3 | 66.8 | 78.8 | 67.1 | 55.8 | 68.8 | 68.2 | 66.8 |  | 53.6 | 63.9 | 55.8 | 61.1 |  |
| **Standard deviation** | 16.4 | 19.6 | 19.2 | 17.5 | 21.9 | 20.0 | 20.7 | 19.7 | 21.9 |  | 21.9 | 19.2 | 21.3 | 19.7 |  |
| **Min-Max** | 12.5-100 | 0-100 | 0-100 | 0-100 | 0-100 | 0-100 | 0-100 | 6.3-100 | 0-100 |  | 0-100 | 0-100 | 0-100 | 0-100 |  |
| **% ceiling** | 1.8 | 6.2 | 7.6 | 26.7 | 15.3 | 3.7 | 16.6 | 6.5 | 14.3 |  | 3.6 | 8.0 | 4.6 | 8.8 |  |
| **% floor** | 0.0 | 0.4 | 0.2 | 0.1 | 0.4 | 0.2 | 0.1 | 0.0 | 0.1 |  | 0.5 | 0.1 | 0.7 | 0.1 |  |
| **Percentiles 25**  **50**  **75** | 48.2  58.3  71.4 | 50.0  58.3  75.0 | 52.1  66.7  79.2 | 66.7  75.0  100 | 50.0  70.8  83.3 | 43.8  53.1  68.8 | 50.0  75.0  81.3 | 56.3  68.8  81.3 | 50.0  70.0  80.0 |  | 39.3  50.0  70.8 | 50.0  62.5  75.0 | 41.7  50.0  70.0 | 50.0  56.3  75.0 |  |
| **Skewness** | 0.29 | 0.12 | -0.05 | -0.44 | -0.22 | 0.21 | -0.14 | -0.64 | -0.18 |  | 0.11 | 0.13 | 0.12 | 0.33 |  |

a Percentage of respondents who completed every item in the scale ASP: Access to primary care INF: Listening abilities and information provided by doctors

b Percentage of respondents who completed at least 50% of items in each scale ASS: Access to secondary care ORG: Organisation and follow-up of medical care provision

c Correlation between items and hypothesized scales, corrected for overlap COMG: Competence and communication skills of primary care doctors PSY: Psychological support

d Internal consistency reliability (Cronbach’s α) COMPS: Competence of secondary care doctors ENV: Material environment

COMMS: Communication skills of secondary care doctors

PC: Choice among doctors

QH: Human qualities of doctors

SG: Global satisfaction

CM: Cover for medical expenses
