# Supplementary material for: Satisfaction with care among patients with non-metastatic breast cancer: development and first steps of validation of the REPERES-60 questionnaire
Source: BMC Cancer. 2007 Jul 16;7:129. doi: 10.1186/1471-2407-7-129 (PMC1933545; doi:10.1186/1471-2407-7-129)
Supplement: Additional file 5 — Discriminant validity – Extreme group comparisons of mean scores (standard deviations) for the thirteen scales of the REPERES-60 questionnaire. [file 1471-2407-7-129-S5.doc]

Discriminant validity - Extreme group comparisons of mean scores (standard deviations) for the thirteen scales of the REPERES-60 questionnaire

| **Dimensions of the**  **REPERES-60 questionnaire** | **Access to primary care** | | |  | **Access to secondary care** | | |  | **Competence and communication skills of primary care doctors** | | |  | **Competence of secondary care doctors** | | | **Communication of secondary care doctors** | | |
| --- | --- | --- | --- | --- | --- | --- | --- | --- | --- | --- | --- | --- | --- | --- | --- | --- | --- | --- |
|  | N | Mean (std) | p-value |  | N | Mean (std) | p-value |  | N | Mean (std) | p-value |  | N | Mean (std) | p-value | N | Mean (std) | p-value |
| **Age** |  |  |  |  |  |  |  |  |  |  |  |  |  |  |  |  |  |  |
| Less than 58 | 400 | 58.9 (16.0) | **0.0050** |  | 402 | 56.6 (19.4) | **< 0.0001** |  | 402 | 65.0 (19.2) | **0.0064** |  | 403 | 78.3 (17.3) | 0.3432 | 404 | 65.0 (22.3) | **0.0060** |
| 58 or above | 410 | 62.1 (16.7) |  |  | 393 | 62.0 (19.5) |  |  | 410 | 68.7 (18.9) |  |  | 409 | 79.4 (17.7) |  | 408 | 69.3 (21.5) |  |
| **Education** |  |  |  |  |  |  |  |  |  |  |  |  |  |  |  |  |  |  |
| Primary and secondary | 543 | 60.8 (16.2) | **0.3813** |  | 529 | 60.3 (19.0) | 0.0128 |  | 544 | 67.6 (18.6) | 0.0549 |  | 544 | 78.9 (17.5) | **0.8971** | 542 | 68.3 (21.4) | **0.0417** |
| Higher education | 246 | 59.4 (16.4) |  |  | 244 | 56.5 (20.1) |  |  | 246 | 64.8 (20.0) |  |  | 246 | 78.8 (17.8) |  | 248 | 64.6 (23.1) |  |
| **Health status** |  |  |  |  |  |  |  |  |  |  |  |  |  |  |  |  |  |  |
| Score < 5 (item 29 of QLQ-C30) | 310 | 58.8 (16.7) | **0.0279** |  | 306 | 56.7 (19.0) | **0.0017** |  | 308 | 64.0 (18.6) | **0.0023** |  | 307 | 76.0 (17.7) | **0.0002** | 309 | 63.4 (22.3) | **0.0005** |
| Score ≥ 5 (item 29 of QLQ-C30) | 500 | 61.5 (16.2) |  |  | 489 | 60.9 (19.8) |  |  | 504 | 68.6 (19.3) |  |  | 505 | 80.5 (17.2) |  | 503 | 69.5 (21.4) |  |
| **Experienced problems of communication in the announcement of the diagnosis** |  |  |  |  |  |  |  |  |  |  |  |  |  |  |  |  |  |  |
| No | 588 | 61.7 (16.4) | **0.0010** |  | 575 | 60.8 (19.5) | **0.0010** |  | 588 | 68.6 (19.0) | **< 0.0001** |  | 592 | 79.7 (17.2) | **0.0141** | 588 | 70.0 (21.3) | **< 0.0001** |
| Yes | 222 | 57.2 (16.1) |  |  | 220 | 55.4 (19.3) |  |  | 224 | 62.3 (19.0) |  |  | 220 | 76.4 (18.3) |  | 224 | 59.7 (22.0) |  |

Significant associations (p < 0.05) are outlined in bold.

For all scales, a higher value represents more satisfaction.

| **Dimensions of the** REPERES-60 questionnaire | **Choice among doctors** | | |  | **Human qualities of doctors** | | |  | **Global satisfaction** | | |  | **Cover for medical expenses** | | |
| --- | --- | --- | --- | --- | --- | --- | --- | --- | --- | --- | --- | --- | --- | --- | --- |
|  | N | Mean (std) | p-value |  | N | Mean (std) | p-value |  | N | Mean (std) | p-value |  | N | Mean (std) | p-value |
| **Age** |  |  |  |  |  |  |  |  |  |  |  |  |  |  |  |
| Less than 58 | 404 | 53.1 (20.3) | **< 0.0001** |  | 404 | 65.5 (21.1) | **< 0.0001** |  | 399 | 63.4 (20.9) | **< 0.0001** |  | 397 | 64.7 (22.2) | **0.0085** |
| 58 or above | 398 | 58.6 (19.2) |  |  | 409 | 72.0 (19.7) |  |  | 403 | 73.0 (17.3) |  |  | 393 | 68.8 (21.5) |  |
| **Education** |  |  |  |  |  |  |  |  |  |  |  |  |  |  |  |
| Primary and secondary | 533 | 57.2 (19.2) | **0.0003** |  | 544 | 70.5 (20.1) | **0.0001** |  | 536 | 70.5 (18.6) | **<0.0001** |  | 528 | 66.9 (21.7) | 0.8164 |
| Higher education | 247 | 52.3 (21.0) |  |  | 247 | 64.5 (21.1) |  |  | 245 | 63.3 (21.5) |  |  | 241 | 66.3 (22.7) |  |
| **Health status** |  |  |  |  |  |  |  |  |  |  |  |  |  |  |  |
| Score < 5 (item 29 of QLQ-C30) | 303 | 54.2 (19.9) | 0.0819 |  | 309 | 64.4 (21.3) | **<0.0001** |  | 306 | 62.6 (21.4) | **<0.0001** |  | 302 | 64.0 (22.7) | **0.0080** |
| Score ≥ 5 (item 29 of QLQ-C30) | 499 | 56.8 (19.9) |  |  | 504 | 71.5 (19.8) |  |  | 496 | 71.7 (17.8) |  |  | 488 | 68.5 (21.3) |  |
| **Experienced problems of communication in the announcement of the diagnosis** |  |  |  |  |  |  |  |  |  |  |  |  |  |  |  |
| No | 582 | 57.1 (19.9) | **0.0122** |  | 590 | 70.8 (20.2) | **< 0.0001** |  | 581 | 70.8 (18.7) | **<0.0001** |  | 572 | 67.4 (21.8) | 0.2371 |
| Yes | 220 | 52.3 (19.8) |  |  | 223 | 63.5 (21.1) |  |  | 221 | 61.4 (20.7) |  |  | 218 | 65.0 (22.2) |  |

Significant associations (p < 0.05) are outlined in bold.

For all scales, a higher value represents more satisfaction.

Item 29 – QLQ-C30: how would you rate your overall health during the past week ? Very poor (1) to excellent (7)

| **Dimensions of the**  **REPERES-60 questionnaire** | **Listening abilities and information provided by doctors** | | |  | **Organisation and follow-up of medical care provision** | | |  | **Psychological support** | | |  | **Material environment** | | |
| --- | --- | --- | --- | --- | --- | --- | --- | --- | --- | --- | --- | --- | --- | --- | --- |
|  | N | Mean (std) | p-value |  | N | Mean (std) | p-value |  | N | Mean (std) | p-value |  | N | Mean (std) | p-value |
| **Age** |  |  |  |  |  |  |  |  |  |  |  |  |  |  |  |
| Less than 58 | 402 | 50.96 (22.15) | **0.0001** |  | 398 | 62.77 (19.60) | 0.0785 |  | 396 | 54.30 (20.92) | **0.0321** |  | 400 | 58.50 (19.83) | **< 0.0001** |
| 58 or above | 394 | 56.23 (21.46) |  |  | 369 | 65.06 (18.74) |  |  | 339 | 57.49 (21.57) |  |  | 404 | 63.66 (19.28) |  |
| **Education** |  |  |  |  |  |  |  |  |  |  |  |  |  |  |  |
| Primary and secondary | 528 | 55.22 (21.33) | **0.0002** |  | 503 | 64.32 (19.30) | 0.2369 |  | 481 | 56.33 (20.53) | 0.0952 |  | 536 | 62.02 (19.17) | **0.0052** |
| Higher education | 247 | 49.84 (22.36) |  |  | 242 | 62.51 (18.70) |  |  | 234 | 54.35 (22.26) |  |  | 246 | 58.41 (20.29) |  |
| **Health status** |  |  |  |  |  |  |  |  |  |  |  |  |  |  |  |
| Score < 5 (item 29 of QLQ-C30) | 310 | 48.23 (20.29) | **< 0.0001** |  | 295 | 59.46 (19.11) | **< 0.0001** |  | 289 | 50.70 (20.87) | **< 0.0001** |  | 309 | 58.89 (19.25) | **0.0078** |
| Score ≥ 5 (item 29 of QLQ-C30) | 493 | 56.84 (22.32) |  |  | 472 | 66.63 (18.79) |  |  | 446 | 59.05 (20.90) |  |  | 495 | 62.47 (19.89) |  |
| **Experienced problems of communication in the announcement of the diagnosis** |  |  |  |  |  |  |  |  |  |  |  |  |  |  |  |
| No | 575 | 56.74 (21.34) | **< 0.0001** |  | 553 | 65.85 (19.17) | **< 0.0001** |  | 526 | 58.22 (20.67) | **< 0.0001** |  | 583 | 62.27 (19.84) | **0.0162** |
| Yes | 221 | 45.28 (21.41) |  |  | 214 | 58.76 (18.43) |  |  | 209 | 49.60 (21.56) |  |  | 221 | 57.98 (19.06) |  |

Significant associations (p < 0.05) are outlined in bold.

For all scales, a higher value represents more satisfaction.

Item 29 – QLQ-C30: how would you rate your overall health during the past week ? Very poor (1) to excellent (7)
