# Supplementary material for: Satisfaction with care among patients with non-metastatic breast cancer: development and first steps of validation of the REPERES-60 questionnaire
Source: BMC Cancer. 2007 Jul 16;7:129. doi: 10.1186/1471-2407-7-129 (PMC1933545; doi:10.1186/1471-2407-7-129)
Supplement: Additional file 6 — REPERES-60 questionnaire (the translation is provided solely for the purpose of informing on item content, and is not a validated measure). [file 1471-2407-7-129-S6.doc]

**REPERES-60 questionnaire -** This translation is provided solely for the purpose of informing on item content, and is not a validated measure**.**

The following statements are about your **medical care**.

Please give your **opinion** on the following points, referring to **your own experience** (Choose only one response for each statement).

**IMPORTANT. If you are not sure how to answer, please choose the response that is closest to your situation. For the questions mentioning several types of doctor (« General Pratitionner (GP) and/or gynaeocologist », « oncologist, surgeon »), please tick only one box, choosing the most appropriate answer.**

| *Access to primary and secondary care* | Bad | Fair | Good | Very good | Excellent |
| --- | --- | --- | --- | --- | --- |
| 1. Hours when your doctor’s office (GP and/or gynaecologist) is open | 🞏 | 🞏 | 🞏 | 🞏 | 🞏 |
| 1. Access to care by a specialist (oncologist, surgeon) | 🞏 | 🞏 | 🞏 | 🞏 | 🞏 |
| 1. Access to hospital care | 🞏 | 🞏 | 🞏 | 🞏 | 🞏 |
| 1. Access to medical care in an emergency | 🞏 | 🞏 | 🞏 | 🞏 | 🞏 |
| 1. How easy it is to get an appointment with your GP and/or gynaecologist by phone | 🞏 | 🞏 | 🞏 | 🞏 | 🞏 |
| 1. Length of time you have to wait in your GP’s and/or gynaecologist’s surgery | 🞏 | 🞏 | 🞏 | 🞏 | 🞏 |
| 1. Length of time you have to wait between the day you make an appointment and the day of consultation with your GP and/or gynaecologist | 🞏 | 🞏 | 🞏 | 🞏 | 🞏 |
| 1. Availability of medical information or advice over the phone from your GP and/or gynaecologist | 🞏 | 🞏 | 🞏 | 🞏 | 🞏 |
| 1. How easy it is to see a doctor whenever you need to (GP and/or gynaecologist) | 🞏 | 🞏 | 🞏 | 🞏 | 🞏 |
| 1. Convenience of access to the GP's and/or gynaecologist’s surgery | 🞏 | 🞏 | 🞏 | 🞏 | 🞏 |

| *competence of your DOCTORS Before your treatment* | Bad | Fair | Good | Very good | Excellent |
| --- | --- | --- | --- | --- | --- |
| 1. The care taken by the GPs and/or gynaecologists you know in examining you and the accuracy of their diagnoses | 🞏 | 🞏 | 🞏 | 🞏 | 🞏 |
| 1. Skill and experience of the GPs and/or gynaecologists you know | 🞏 | 🞏 | 🞏 | 🞏 | 🞏 |
| 1. Thoroughness of GPs and/or gynaecologists you know in choosing treatment | 🞏 | 🞏 | 🞏 | 🞏 | 🞏 |
| *competence of your DOCTORS for your treatment* |  |  |  |  |  |
|  |  |  |  |  |
| 1. The care taken by the (medical) specialists (oncologists, surgeons) you know in examining you and the accuracy of their diagnoses | 🞏 | 🞏 | 🞏 | 🞏 | 🞏 |
| 1. Competence and experience of the specialists (oncologists, surgeons) you know | 🞏 | 🞏 | 🞏 | 🞏 | 🞏 |
| 1. Thoroughness of the specialists (oncologists, surgeons) you know in choosing treatment | 🞏 | 🞏 | 🞏 | 🞏 | 🞏 |

| *Attention paid (to you) and information provided by doctors* | Bad | Fair | Good | Very good | Excellent |
| --- | --- | --- | --- | --- | --- |
| 1. The explanations provided by the GPs and/or gynaecologists you know on medical procedures and tests | 🞏 | 🞏 | 🞏 | 🞏 | 🞏 |
| 1. The attention paid by GPs and/or gynaecologists you know to what you say | 🞏 | 🞏 | 🞏 | 🞏 | 🞏 |
| 1. Advice on preventative measures you receive from GPs and/or gynaecologists to stay healthy | 🞏 | 🞏 | 🞏 | 🞏 | 🞏 |
| 1. The explanations provided by the specialists (oncologists, surgeons) you know on medical procedures and tests | 🞏 | 🞏 | 🞏 | 🞏 | 🞏 |
| 1. The attention paid by specialists (oncologists, surgeons) you know to what you say | 🞏 | 🞏 | 🞏 | 🞏 | 🞏 |
| 1. Advice on preventative measures you receive from specialists (oncologists, surgeons) to stay healthy | 🞏 | 🞏 | 🞏 | 🞏 | 🞏 |

| *Choice among different doctors* | Bad | Fair | Good | Very good | Excellent |
| --- | --- | --- | --- | --- | --- |
| 1. The number of GPs and/or gynaecologists you can consult | 🞏 | 🞏 | 🞏 | 🞏 | 🞏 |
| 1. How easy it is to consult the GP and/or the gynaecologist of you choice | 🞏 | 🞏 | 🞏 | 🞏 | 🞏 |
| 1. The number of specialists (oncologists, surgeons) you can consult | 🞏 | 🞏 | 🞏 | 🞏 | 🞏 |
| 1. How easy it is to consult the specialist (oncologist, surgeon) of your choice | 🞏 | 🞏 | 🞏 | 🞏 | 🞏 |

| *Human qualities shown by doctors* | Bad | Fair | Good | Very good | Excellent |
| --- | --- | --- | --- | --- | --- |
| 1. Kindness (friendliness) and courtesy of doctors | 🞏 | 🞏 | 🞏 | 🞏 | 🞏 |
| 1. Interest taken by doctors in you and your health problems | 🞏 | 🞏 | 🞏 | 🞏 | 🞏 |
| 1. Respect shown to you by doctors and attention to privacy | 🞏 | 🞏 | 🞏 | 🞏 | 🞏 |
| 1. The ability of doctors to reassure you and give you support | 🞏 | 🞏 | 🞏 | 🞏 | 🞏 |

*Satisfaction overall*

Below you will find remarks made by patients concerning the care they have received. Please read them carefully. Even if some sentences seem similar, please answer each separately.

|  | Completely agree | Agree generally | No marked opinion | Do not really agree | Do not agree at all |
| --- | --- | --- | --- | --- | --- |
| 1. I am very satisfied with the care I receive | 🞏 | 🞏 | 🞏 | 🞏 | 🞏 |
| 1. Some things in the care I receive could be better | 🞏 | 🞏 | 🞏 | 🞏 | 🞏 |
| 1. The care I receive is practically perfect | 🞏 | 🞏 | 🞏 | 🞏 | 🞏 |
| 1. I am dissatisfied with some things in the care I receive | 🞏 | 🞏 | 🞏 | 🞏 | 🞏 |

The next part of this questionnaire concerns your opinion on more specific aspects of how your medical and social needs are catered for.

*Cover for medical expenses*

The following statements concern your health cover, that is to say what is paid for by your social security insurance, and your complementary health insurance if you have one. Please give your opinion on each of the following statements.

|  | Bad | Fair | Good | Very good | Excellent |
| --- | --- | --- | --- | --- | --- |
| 1. The ability of your health cover to compensate for medical expenses and loss of income | 🞏 | 🞏 | 🞏 | 🞏 | 🞏 |
| 1. The range of costs that are reimbursed | 🞏 | 🞏 | 🞏 | 🞏 | 🞏 |
| 1. The reimbursement of your consultation fees | 🞏 | 🞏 | 🞏 | 🞏 | 🞏 |
| 1. The reimbursement of your hospital expenses | 🞏 | 🞏 | 🞏 | 🞏 | 🞏 |
| 1. The reimbursement of your expenditure for medication | 🞏 | 🞏 | 🞏 | 🞏 | 🞏 |

Your opinion of how your medical and social needs are catered for.

| *Listening abilities and information provided by doctors* | Bad | Fair | Good | Very good | Excellent | Not concerned |
| --- | --- | --- | --- | --- | --- | --- |
| 1. The explanations given you to help you prepare for the consequences of surgery | 🞏 | 🞏 | 🞏 | 🞏 | 🞏 |  |
| 1. The information given you on your treatment as a whole | 🞏 | 🞏 | 🞏 | 🞏 | 🞏 |  |
| 1. The information given you on the consequences of the illness | 🞏 | 🞏 | 🞏 | 🞏 | 🞏 |  |
| 1. The information given you on side effects of the treatment | 🞏 | 🞏 | 🞏 | 🞏 | 🞏 |  |
| 1. The information given you on pain management | 🞏 | 🞏 | 🞏 | 🞏 | 🞏 |  |
| 1. The information given you on the possibilities for breast reconstruction | 🞏 | 🞏 | 🞏 | 🞏 | 🞏 | 🞏 |
| 1. The quality of the information received about your disease | 🞏 | 🞏 | 🞏 | 🞏 | 🞏 |  |

| *Organisation and follow-up of medical care provision* | Bad | Fair | Good | Very good | Excellent | Not concerned |
| --- | --- | --- | --- | --- | --- | --- |
| 1. Communication overall among all the different doctors who have cared for you since your diagnosis | 🞏 | 🞏 | 🞏 | 🞏 | 🞏 |  |
| 1. Time-lapse before receiving surgery (if applicable) | 🞏 | 🞏 | 🞏 | 🞏 | 🞏 | 🞏 |
| 1. Time-lapse before receiving chemotherapy (if applicable) | 🞏 | 🞏 | 🞏 | 🞏 | 🞏 | 🞏 |
| 1. Time-lapse before receiving radiotherapy (if applicable) | 🞏 | 🞏 | 🞏 | 🞏 | 🞏 | 🞏 |
| 1. Time taken by the specialist doctors to inform your GP | 🞏 | 🞏 | 🞏 | 🞏 | 🞏 |  |

| *Psychological support* | Bad | Fair | Good | Very good | Excellent |
| --- | --- | --- | --- | --- | --- |
| 1. In case of hospitalisation, the opportunities to talk to someone about your health problems when you needed to (people close to you, an association, medical and non-medical staff) | 🞏 | 🞏 | 🞏 | 🞏 | 🞏 |
| 1. When at home, the opportunities to talk to someone about your health problems when you needed to (people close to you, an association, medical and non-medical staff) | 🞏 | 🞏 | 🞏 | 🞏 | 🞏 |
| 1. The psychological support that the doctors provided you with | 🞏 | 🞏 | 🞏 | 🞏 | 🞏 |
| 1. The psychological support that the nurses provided you with | 🞏 | 🞏 | 🞏 | 🞏 | 🞏 |
| 1. The assistance provided by the medical world throughout your care | 🞏 | 🞏 | 🞏 | 🞏 | 🞏 |

| *Material environment* | Bad | Fair | Good | Very good | Excellent |
| --- | --- | --- | --- | --- | --- |
| 1. The quality of the consultation premises you visited (quietness, atmosphere etc) | 🞏 | 🞏 | 🞏 | 🞏 | 🞏 |
| 1. The quality of the hospital wards you have been in (quietness, atmosphere etc) | 🞏 | 🞏 | 🞏 | 🞏 | 🞏 |
| 1. The respect for your privacy in the consultation premises you visited | 🞏 | 🞏 | 🞏 | 🞏 | 🞏 |
| 1. The respect for you privacy on hospital wards that you have been in | 🞏 | 🞏 | 🞏 | 🞏 | 🞏 |
